# The Role of Serotonin beyond the Central Nervous System during Embryogenesis

**DOI:** 10.3389/fncel.2017.00074

**Published:** 2017-03-13

**Authors:** Junhua Lv, Feng Liu

**Affiliations:** ^1^State Key Laboratory of Membrane Biology, Institute of Zoology, Chinese Academy of SciencesBeijing, China; ^2^University of Chinese Academy of SciencesBeijing, China

**Keywords:** serotonin, hematopoietic system, inflammatory response, tissue regeneration, embryogenesis

## Abstract

Serotonin, or 5-hydroxytryptamine (5-HT), is a well-known neurotransmitter that plays vital roles in neural activities and social behaviors. Clinically, deficiency of serotonin is linked with many psychiatric disorders. Interestingly, a large proportion of serotonin is also produced outside the central nervous system (CNS). There is increasing evidence demonstrating important roles of serotonin in the peripheral tissues. Here, we will describe the multiple biological functions of serotonin in hematopoietic system, such as development of hematopoietic stem and progenitor cells (HSPCs), differentiation of hematopoietic cells, maintenance of vascular system, and relationship with hematological diseases. The roles of serotonin in inflammatory responses mediated by hematopoietic cells as well as in liver regeneration are also discussed. Our recent understandings of the impact of serotonin on hematopoietic system, immune responses, and tissue regeneration support utilization of serotonin as a potential therapeutic target for the treatment of hematological diseases and organ repair in clinic.

## Introduction

As one of the most classical monoamine neurotransmitters and hormones in the central nervous system (CNS) and peripheral tissues, serotonin (also called 5-hydroxytryptamine [5-HT]) has been discovered for nearly 70 years. Serotonin was isolated from the serum for the first time in 1948 (Rapport et al., 1948). Soon after, enteramine was isolated and characterized from the gut enterochromaffin cells (Erspamer and Boretti, 1951), and finally identified to be the same as serotonin discovered in the serum. Although serotonin is abundant in the peripheral tissues, much attention has been focused on its function in the CNS.

Serotonin has been extensively studied in the CNS for its essential role in embryos and adults. In the CNS of mice, serotonergic neurons are specified and matured at embryonic day (E) 10.5 and E12.5, respectively, and finally locate in the hindbrain of adult (Goridis and Rohrer, 2002; Pattyn et al., 2004). According to their locations, serotonergic neurons are grouped into nine clusters, with clusters B1–B3 and clusters B4–B9 present in the caudal and rostral part of the hindbrain, respectively (Halliday et al., 1995). The generation of serotonergic neurons is tightly controlled by a complex of signaling and gene regulatory network, such as sonic hedgehog (Shh) signaling and the Nkx2-2-Lmx1b-Pet1 cascade (Ding et al., 2003). Animals including humans need serotonin secreted from the serotonergic neurons to regulate mood, appetite and sleep. Serotonin also plays roles in cognition, such as memory and learning (Berridge et al., 2009). The feelings of well-being and happiness are related with serotonin (Liu et al., 2014; Li et al., 2016). Furthermore, several social behaviors have been reported to be regulated by serotonin. For example, in adult male mice, the level of serotonin in the brain controls sexual preference (Liu et al., 2011). Its deficiency certainly leads to many psychiatric disorders. Humans with low level of serotonin are more prone to depression, suicide and violence. An obvious example is that the rate of serotonin synthesis in the brain of females is only about half of that in males, which may explain why the females have a higher probability of distressing depressive disorders (Nishizawa et al., 1997). Similarly, decrease in serotonin synthesis and high rate of depressive disorders can also be found in aged human beings. In clinics, serotonin attracts extensive attention for its therapeutic effect on treatment of depression, schizophrenia and anxiety (Owens and Nemeroff, 1994; Hirschfeld, 2000).

Notably, only about 1%−2% of total amount of serotonin is produced by serotonergic neurons in the brain, whereas 90% of serotonin is detected to be secreted from the enterochromaffin cells of gastrointestinal (GI) tract (Gershon and Tack, 2007). There is also a considerable number of serotonin receptors expressed in various peripheral organs. These observations suggest that serotonin is not only an important neurotransmitter in the CNS, but also may exert its effects through its receptors specifically expressed in different peripheral tissues.

The goal of this minireview article is to discuss the extensive roles of serotonin in the peripheral tissues, especially in hematopoietic system. We will focus on its roles in promoting hematopoietic stem and progenitor cell (HSPC) development in cell autonomous and non-cell autonomous manners and regulating erythropoiesis and megakaryocytopoiesis. A brief discussion of the effects of serotonin on immune response and tissue regeneration will also be included.

## Synthesis and Metabolism of Serotonin

**Figure 1:**
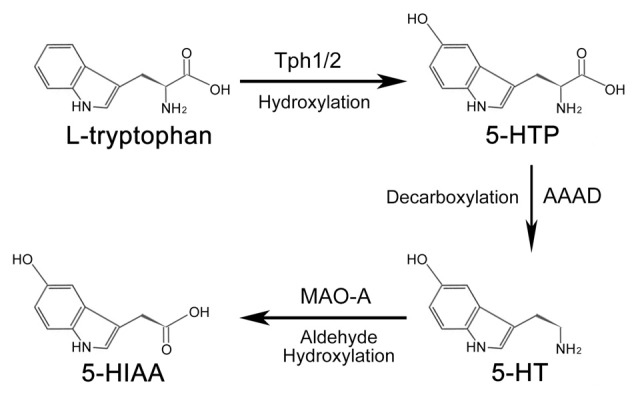
**Synthesis and metabolism process of serotonin.** In animals, serotonin is synthesized from amino acids L-tryptophan. Under the hydroxylation of tryptophan hydroxylase (Tph), L-tryptophan is converted into 5-hydroxytryptophan (5-HTP), which is subsequently catalyzed into serotonin by aromatic amino acid decarboxylase (AAAD). Tph1 and Tph2 are two forms of Tph. Once the biological function of serotonin is accomplished, it is finally metabolized into 5-hydroxyindole acetic acid (5-HIAA) to be removed from the body.

In both the CNS and peripheral tissues of animals, the amino acid L-tryptophan is the primary source of serotonin (Figure 1). Under the catalization of tryptophan hydroxylase (TPH), a hydroxyl is added to L-tryptophan to form 5-hydroxytryptophan (5-HTP). Conversion of L-tryptophan to 5-HTP is the rate-limiting step in the synthesis of serotonin (Lovenberg et al., 1967; Ichiyama et al., 1970). There are two forms of TPH—broadly-expressed Tph1 and CNS-enriched Tph2 (Côté et al., 2003). Although mainly expressed in the peripheral tissues (e.g., gut, skin, pineal and gland), Tph1 is also reported to be present in the CNS (Zill et al., 2009). Similarly, Tph2 is also detected in the aorta-gonad-mesonephros (AGM) region of zebrafish and mouse embryos by RNA-seq and immunofluorescence assay (Zhang et al., 2015; Lv et al., 2017). 5-HTP is subsequently converted into serotonin through the decarboxylation process mediated by aromatic amino acid decarboxylase (AAAD; Lovenberg et al., 1967; Ichiyama et al., 1970). Other than converting 5-HTP into serotonin, AAAD can also participate in other decarboxylation reactions, such as converting L-dopa into dopamine in dopaminergic, noradrenergic and adrenergic neurons (Christie et al., 2014).

Serotonin activates the intracellular signaling cascade through its 15 receptors, which are classified into seven families (Hannon and Hoyer, 2008). Once the biological function of serotonin is accomplished, the metabolism of serotonin would be carried out by the outer mitochondrial membrane enzyme monoamine oxidase A (MAO-A) to generate 5-hydroxyindole acetic acid (5-HIAA). 5-HIAA is the metabolite of serotonin without any biological activity (Shih et al., 1999; Singh et al., 1999), which is excreted out of the body by the kidney. The metabolism process of serotonin is mainly processed in the liver.

## Serotonin and Hematopoietic System

### Serotonin and HSPCs

**Figure 2:**
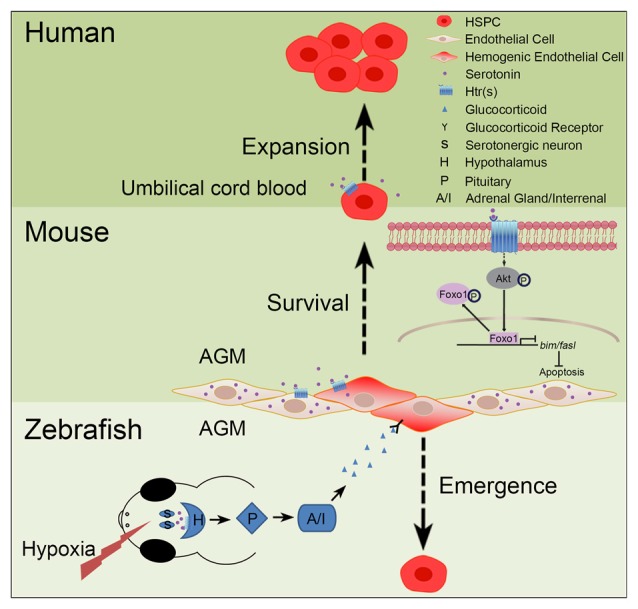
**Role of serotonin in hematopoiesis.** In zebrafish and mouse embryos, serotonin has been demonstrated to control the development of hematopoietic stem and progenitor cells (HSPCs), but through different mechanisms. In zebrafish, stresses, such as hypoxia, can induce the release of serotonin from serotonergic neurons in the central nervous system (CNS); through the receptor(s) on hypothalamus, serotonin can activate the hypothalamic-pituitary-adrenal/interregnal (HPA/I) axis to promote the production of glucocorticoid in the aorta-gonad-mesonephros (AGM) and accelerate the formation of HSPCs. In contrast, in the AGM of mouse embryos, serotonin can be directly produced by the endothelial cells to maintain the survival of HSPCs. This process is regulated, through serotonin receptors expressed on HSPCs, by inhibiting the pro-apoptotic pathway mediated by the AKT-Foxo1 signaling. In humans, serotonin can also promote the expansion of umbilical cord blood CD34^+^ cells *in vitro* and *ex vivo*.

Increasing evidence has implicated the relationship between serotonin and HSPCs (Figure 2). Yang et al. (2007) have reported that addition of serotonin enhances the colony formation ability of human umbilical cord blood CD34^+^ cells and increases the reconstitution level of CD45^+^ cells in the bone marrow of irradiated immunodeficient nonobese diabetic-severe combined immunodeficient (NOD/SCID) mice. Our recent study has shown that serotonin, which is synthesized in the endothelial cells of AGM, promotes the survival of HSPCs in the intra-aortic hematopoietic clusters of mouse embryos (Lv et al., 2017). These findings support that serotonin can act as an endogenous factor to regulate the development of HSPCs during embryogenesis. Similarly, a study in zebrafish system has also demonstrated that treatment of the embryos with serotonin increases the formation of HSPCs in the AGM (Kwan et al., 2016). Mechanistically, serotonin is identified to regulate the population of HSPCs in zebrafish embryos through the production of cortisol controlled by the hypothalamic-pituitary-adrenal/interregnal (HPA/I) axis. However, whether serotonin can regulate HSPC development through HPA/I-mediated signaling in mammals and whether serotonin exerts its effect cell-autonomously in zebrafish still need further investigation.

### Serotonin and Differentiation of Hematopoietic Cells

The regulation of serotonin on differentiated hematopoietic cells has also been reported. Serotonin is an important regulator for the survival of red blood cells (RBCs) *in vivo* and that erythropoiesis is obviously impaired in mice with peripheral serotonin deficiency (Amireault et al., 2011). Further analysis reveals that serotonin in the microenvironment acts as an antioxidant extrinsic effector to protect RBCs from senescence (Amireault et al., 2013). Serotonin has also been shown to bind onto the surface of platelets and the conjugation of serotonin to the procoagulant proteins could stimulate platelet activation (Dale et al., 2002). Platelet-derived serotonin influences the differentiation of monocytes into dendritic cells (DCs) and impairs the differentiation of DCs to T cells (Katoh et al., 2006).

Serotonin has also been identified to play important roles in megakaryocytopoiesis. Although most of serotonin is stored in the platelets, a small quantity of serotonin can also be stored in the megakaryocytes (MKs). MKs are the progenitors of platelets and the only cells to take up serotonin in the bone marrow (Liu and Yang, 2006). Serotonin receptors have been reported to be expressed on the surface of most MKs. The *in vitro* study shows that serotonin can enhance MK colony formation ability and the mitogenic effect of serotonin on megakaryocytopoiesis may be mediated through the Htr2 receptor (Yang et al., 1996). A recent study has demonstrated that Htr2b is expressed in MKs and that serotonin mediated by Htr2b can enhance the proliferation and inhibit the apoptosis of MKs. This study also shows that serotonin can activate ERK signaling and affect F-actin reorganization to promote megakaryopoiesis and proplatelet formation (Ye et al., 2014).

### Serotonin and Vascular Maintenance

The vast majority of serotonin is secreted by the enterochromaffin cells of GI tract to control the movement of intestine (Gershon and Tack, 2007). Platelets themselves cannot synthesize serotonin, due to a lack of enzymes responsible for serotonin synthesis. Instead, circulating platelets actively take up the serotonin released into the blood from the tissues (Vanhoutte, 1991; Ni and Watts, 2006). The reabsorbed serotonin in the blood can play important roles in vascular biology, including platelet activation, hemostasis and vascular endothelial cell and smooth muscle cell proliferation.

Under normal condition, platelets are kept balanced between quiescence and activation. Through binding onto the surface of platelets and conjugation to the adhesion and procoagulant proteins, serotonin can stimulate the activation of platelets (Dale et al., 2002). Another study also shows that platelet activation is mediated by the covalent cross-linkage of serotonin with small G proteins and the activation of G protein-dependent downstream signaling pathways (Walther et al., 2003).

During hemostasis, the activation of platelets is an important process and the “golden standard” assay of platelets activation is to detect the release of serotonin (Gobbi et al., 2003). At the injury site of blood vessels, serotonin is secreted from the platelets bound with the receptors expressed on the damaged vessels and acts as a vasoconstrictor to block bleeding (Kaumann and Levy, 2006). Selective serotonin reuptake inhibitors (SSRIs) treatment can inhibit the storage of serotonin in the platelets and increase the bleeding time. Moreover, platelet aggregation would also be decreased in serotonin transporter knockout mice (Carneiro et al., 2008).

In addition, the receptors of serotonin, such as Htr1b, Htr2a, Htr2b, Htr4 and Htr7, are all found to be expressed in vascular endothelial cells and smooth muscle cells (Kaumann and Levy, 2006; Monassier et al., 2010). These results indicate the role of serotonin in vasculature. Serotonin is reported to possess mitogenic effect on the vascular endothelial cells (Pakala et al., 1994). Mediated by the receptor Htr1b, serotonin can stimulate angiogenesis through the AKT-eNOS pathway in diabetic mice (Iwabayashi et al., 2012). Similarly, other reports show that serotonin acts as an angiogenic factor to induce endothelial cell proliferation through TR3/Nur77 signaling in mice and Src/PI3K/AKT/mTOR/p70S6K signaling in human, respectively (Zamani and Qu, 2012; Qin et al., 2013). In the absence of Htr2b, the differentiation, proliferation and mobilization of endothelial progenitor cells from the bone marrow are reported to be impaired (Ayme-Dietrich et al., 2016). Furthermore, serotonin can also stimulate the proliferation of vascular smooth muscle cells (Penumatsa et al., 2014).

### Serotonin and Hematological Diseases

As serotonin plays critical roles in the development and differentiation of HSPCs, many hematological diseases are related with its dysregulation. Study in *Tph1*^−/−^ mice demonstrates that serotonin regulates the balance of Th17 cells and T regulatory cells and is involved in arthritis, which is an important autoimmune disease (McAlpine et al., 2016). Elevated level of serotonin is observed in the serum of asthmatic patients, an inflammatory disease of lung (Cazzola and Matera, 2000). Using the mouse model, researchers also show the inhibitory role of Htr2 agonist in allergic asthma (Nau et al., 2015).

## Serotonin and Inflammatory Response

Although platelets are traditionally considered to play vital roles in hemostasis at the site of injury, more evidence has demonstrated that platelets can also act as an immune effector to participate in the inflammation under physiological and pathological conditions (Li et al., 2012; Jenne and Kubes, 2015). A large amount of serotonin is stored in the platelets and serotonin would be released into the blood from platelets upon injury and infection, suggesting that serotonin may play a role in immune response. Many studies have shown the regulatory function of serotonin on differentiated hematopoietic cells during the inflammatory process. In infected tissues, the aggregate of serotonin can protect natural killer (NK) cells from mononuclear phagocytes-induced apoptosis, through scavenging reactive oxygen species (ROS) generated by the myeloperoxidase-H_2_O_2_ system (Betten et al., 2001, 2004). Similarly, serotonin has been reported to efficiently induce interferon-gamma (IFN-γ) production in NK cells (Hellstrand et al., 1993). The mRNA levels and protein release of cytokines, such as interleukin (IL)-1beta, IL-6, IL-8/CXCL8, IL-12p40 and tumor necrosis factor-alpha (TNF-α), can be modulated by serotonin in monocytes through its receptors Htr3, Htr4 and Htr7 during inflammation (Dürk et al., 2005). Furthermore, serotonin can also inhibit apoptosis of monocytes through upregulation of Bcl2 and Mcl1 and therefore maintain the survival of monocytes during chronic inflammation (Soga et al., 2007). Through the receptor Htr7 expressed on naïve T cells, serotonin can induce the activation of ERK and NF-κB signalings and contribute to T cell activation during inflammation (León-Ponte et al., 2007). Studies in mouse bone marrow derived- and human CD34^+^ cell-derived mast cells both have shown that serotonin stimulation could facilitate these cells to adhere to fibronectin and to migrate towards and accumulate at the site of injury through Htr1a (Kushnir-Sukhov et al., 2006).

## Serotonin and Tissue Regeneration

The liver is a unique organ, which possesses the ability to regenerate (Michalopoulos and DeFrances, 1997). Several signaling factors have been shown to contribute to liver regeneration, including cytokines, growth factors, hormones and nuclear receptors (Michalopoulos, 2013). It has been identified that platelets are vital for the regeneration of liver (Lesurtel et al., 2006; Murata et al., 2007, 2008; Takahashi et al., 2013). Among these studies, Lesurtel et al indicate that platelet-derived serotonin can stimulate liver regeneration (Lesurtel et al., 2006). The decrease of serotonin in *Tph1* knockout mice and inhibition of Htr2a and Htr2b, the receptors of serotonin, both impair the process of liver regeneration. The mechanism of serotonin-mediated liver regeneration is still controversial (Lisman and Porte, 2016). On the one hand, serotonin may promote the mitogenic process of hepatocytes in mice after hepatectomy; on the other hand, the decrease in serotonin influences the platelet activation, while the delayed liver regeneration may be a secondary effect of the impaired platelet activation. Since serotonin is known to participate in the platelet activation, the decrease in serotonin would lead to the impairment of platelet response (Dale et al., 2002; Walther et al., 2003).

## Perspectives

Defining the crucial roles of serotonin during hematopoiesis is particularly useful for new design to treat hematological diseases. More than 10 types of functional hematopoietic cells are differentiated from hematopoietic stem cells (HSCs), which also possess the capacity to self-renew. The demand of functional HSCs for transplantation to cure patients with hematological diseases and other malignancies is rapidly increasing in clinics. Although many efforts and progresses have been made to expand umbilical cord blood-derived HSPCs (Boitano et al., 2010; Delaney et al., 2010; Fares et al., 2014; Rentas et al., 2016), obtaining a large amount of functional HSPCs for clinical use is not yet feasible. Recent studies suggest the potential of serotonin in expanding HSPCs. It has been shown that serotonin treatment can enhance the *in vitro* colony formation ability of human umbilical cord blood CD34^+^ cells as well as reconstitution of CD45^+^ cells in NOD/SCID mice (Yang et al., 2007). During embryogenesis, serotonin can also increase the colony formation ability of HSPCs in the AGM of mouse embryos directly, and the HSPC population in zebrafish indirectly (Kwan et al., 2016; Lv et al., 2017). Considering its crucial role in HSPC development and expansion, serotonin might serve as a good target for clinical use. In particular, inhibition of Htr5a, a highly-enriched serotonin receptor in the AGM of mouse embryos, leads to the impairment of colony formation of HSPCs through AKT-Foxo1-mediated apoptotic pathway (Lv et al., 2017). The serotonin receptors can produce an excitatory or inhibitory response. It is of note that many of these receptors have been validated to be the targets of a variety of pharmaceutical drugs, including many well-known antidepressants (Nichols and Nichols, 2008). Therefore, Htr5a is also a potential target to expand HSPCs both *in vitro* and *in vivo*.

In addition, serotonin plays important roles in normal erythropoiesis and megakaryocytopoiesis to generate functional RBCs and platelets. Furthermore, serotonin is also a vital effector for the survival of and cytokine release of NK cells as well as activation of T cells, all of which are essential for immune response. These findings indicate that serotonin is required for the development of functional hematopoietic cells under both physiological and pathological conditions.

There is evidence supporting the stimulatory effects of serotonin in liver regeneration (Lesurtel et al., 2006). However, the underlying mechanism of platelet-derived serotonin to mediate liver regeneration is still unclear. Similarly, the role of platelets in liver regeneration remains partially understood at the molecular level (Lisman et al., 2015). More detailed studies on the roles and mechanisms of serotonin-mediated liver regeneration are required, which would provide insights into designing new therapeutic strategies for clinical liver regeneration.

## Conclusion

Serotonin plays irreplaceable roles in the CNS and its deficiency causes many psychiatric disorders. Interestingly, many unexpected functions of serotonin in the peripheral tissues during embryogenesis have been recognized recently. However, how exactly serotonin plays its role is still not well defined. For example, during HSPC development, both the local action and HPA/I mediated CNS effects of serotonin on the development of HSPCs in mouse and zebrafish embryos have been reported; however the reason for the discrepancy between mouse and zebrafish embryos remains to be addressed. Moreover, how serotonin increases the colony formation ability of human umbilical cord blood CD34^+^ cells and promotes reconstitution of CD45^+^ cells in the bone marrow of NOD/SCID mice at the molecular level are still obscure. A better understanding of the underlying mechanisms of serotonin in the peripheral tissues would facilitate its potential clinical application in the future.

## Author Contributions

JL and FL conceived the project, analyzed the data and wrote the article. Both authors read and approved the final manuscript.

## Conflict of Interest Statement

The authors declare that the research was conducted in the absence of any commercial or financial relationships that could be construed as a potential conflict of interest.
